# Diagnosis of peste des petits ruminants infection in small ruminants through in-house developed Indirect ELISA: Practical considerations

**DOI:** 10.14202/vetworld.2015.443-448

**Published:** 2015-04-07

**Authors:** K. K. Sharma, D. P. Kshirsagar, I. H. Kalyani, D. R. Patel, P. D. Vihol, J. M. Patel

**Affiliations:** 1 Department of Veterinary Microbiology, Vanbandhu College of Veterinary Science and Animal Husbandry, Navsari Agricultural University, Navsari, Gujarat, India; 2 Department of Veterinary Public Health and Epidemiology, Vanbandhu College of Veterinary Science and Animal Husbandry, Navsari Agricultural University, Navsari, Gujarat, India; 3 Department of Veterinary Pathology, Vanbandhu College of Veterinary Science and Animal Husbandry, Navsari Agricultural University, Navsari, Gujarat, India

**Keywords:** agar gel immunodiffusion, competitive ELISA, Goat, indirect ELISA, peste des petits ruminants

## Abstract

**Aim:**

The work was conducted to diagnose peste des petits ruminants (PPR) outbreak through an in house developed indirect ELISA (thereafter referred as iELISA) its comparison with other available diagnostic tests and description of practical considerations in its development, utility and limitations.

**Materials and Methods:**

An outbreak resembled to PPR occurred in two different places of southern Gujarat *viz*. Vapi and Navsari, affecting 622 animals, including both goat (n = 476) and sheep (n = 146). Animals displayed the typical signs of PPR at Vapi; however diarrhea was the inconsistent feature in animals of Navsari. The affection caused morbidity of 100% and mortality were 73.68% (n = 392/532) and 56.67% (n = 51/90) in Vapi and Navsari outbreaks, respectively. Relevant ante mortem and post mortem samples were collected from representative animals. At the outset of the epidemic no kit was available with us, so agar gel immunodiffusion (AGID) was carried out and a commercial ELISA (cELISA) kit was ordered for making diagnosis through antibody demonstration. Meanwhile, an iELISA was developed in house using PPR vaccine as antigen and protein G conjugated HRPO antibody as detector. Histopathology and results of sandwich ELISA were also used to diagnose PPR virus (PPRV) in the outbreak.

**Results:**

The iELISA developed had detected PPRV antibodies in 22/24 samples (91.66%). Significant difference was observed in disease sensitivity pattern of two species by Chi-square test. While AGID failed to detect antibodies in any sample. Results were reconfirmed by comparing with commercially available cELISA kit.

**Conclusion:**

PPR is an economically important disease and for the rapid diagnosis of PPR the in house developed antibody capture iELISA can be a suitable cost effective alternative.

## Introduction

Peste des petits ruminants (PPR) is translated as “plague of small ruminants”, which is caused by an *ss* negative sense ribonucleic acid containing enveloped virus PPR virus (PPRV) which is member of genus morbillivirus of family *Paramyxoviridae* [1]. This is an office international des epizootics (OIE) “list A” disease characterized by respiratory, lymphatic, and alimentary tract affection as the name indicates; it causes heavy morbidity and mortality in small ruminants like goat and sheep [2].

Now, this disease has established as endemic in India and prevalent in almost every part of the country where goat and sheep flocks are reared. Sheep and goat husbandry is restricted to poor and marginal farmers of India as the entry of the virus and resultant morbidity and mortality pose havoc on the livelihood of affected farmers. It has been estimated that this disease alone causes economic loss of 1800 million Indian rupees (approximately US$ 39 million) per year [3]. In endemic areas, PPR is considered to be one of the main constraints to improve productivity of small ruminants [4]. Though clinical signs are suggestive of disease, but clinical picture warrants differentiation of infection from many diseases, particularly with caprine contagious pleuropneumonia and hemorrhagic septicemia [2], which is possible through certain laboratory-based microbiological tests. PPRV infection can be diagnosed through precipitation tests like Agar gel immunodiffusion (AGID), counter immunoelectrophoresis, ELISA (antigen detecting sandwich ELISA (sELISA) or antibody detecting cELISA), polymerase chain reaction (PCR) includes reverse transcription PCR or qRTPCR, cell culture, and virus neutralization test (VNT) [2,5]. All of the methods mentioned above have their own merit and demerits. Precipitation tests are though easy to perform, but they lack the sensitivity and specificity. PCR and cell culture-based methods are very costly and technically demanding, again cell culture methods, including are very time-consuming, which cannot commensurate for field based diagnosis of the acute infections like PPR. ELISA is though not free from limitation but better suited among the candidate tests [6,7]. Two types of ELISA has been employed by various workers for PPR diagnosis; Antigen capturing (sELISA) for antigen detection [8-10] and monoclonal antibody-based competitive ELISA (cELISA) for antibody detection [7,11,12]. Both the types of ELISA are available commercially, but in secondary set up laboratories under tropical conditions, like ours, the cost, shelf life of kit due to deterioration of its heat labile components are major hurdles to remain equip all the time for PPR diagnosis. Whereas, acuteness, associated morbidity, and mortality, as well as poor economic condition of animal owners, warrant rapid diagnosis.

Similar conditions have been described by Balamurgan *et al*. [1], where author suggested going for in house developed Indirect ELISA (iELISA), which is well comparable with commercial cELISA system or VNT. Hence, when an outbreak suggestive of PPR had been investigated, it was confirmed by detection of PPR antibodies in sera of affected animals through an in house developed antibody capture iELISA and results were reconfirmed by comparing with commercially available cELISA kit.

The present communication description is given regarding considerations encountered during its development and thereafter disease confirmation through it.

## Materials and Methods

### Ethical approval

As per CPCSEA guidelines, study involving clinical samples does not require approval of Institute Animal Ethics Committee and the authors were permitted by animal owners for sampling.

### Location, animals and sampling

An outbreak of disease resembled to PPR was occurred in different flocks of small ruminants, maintained for subsistence purpose, in two southern towns of Gujarat state of India *viz*. Navsari and Vapi, in February - March months. Though, this region is known for high humidity and heavy rainfall but that time climate was like to typical springs of India. Where temperature remains around 30°C with moderate humidity and no rainfall was recorded at that time.

**Figure-1:**
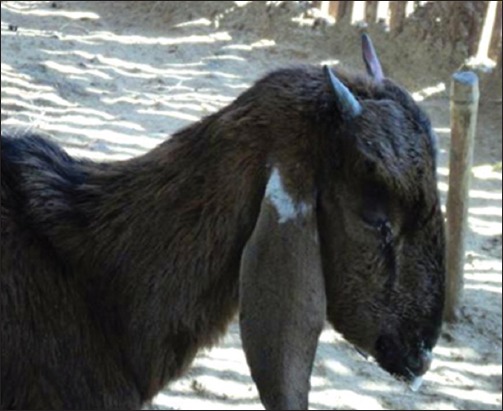
Lacrimation and mouth-frothing in goats from the outbreak area.

**Figure-2:**
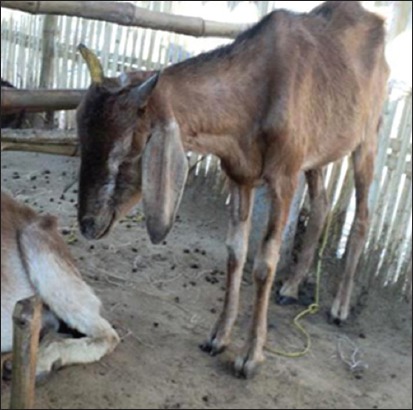
Respiratory distress in goats from the outbreak area.

**Figure-3:**
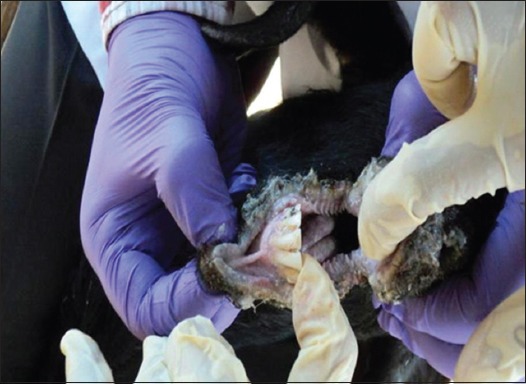
Mouth erosions in goats from the outbreak area.

The flock of Vapi was comprised of both sheep and goat (n = 532, with 140 survivors only), whereas in Navsari, flocks contained goat only (n = 90, with 41 survivors). As a predisposing factor, transportation stress was associated with all the animals. The affected animals showed typical signs of PPR, including high fever, erosion in mouth, stomatitis, mucopurulent nasal discharge, sticky eyes, scab on mouth and lips, labored breathing, signs of pneumonia, and coughing as shown in Figures-1-3. At Vapi, it resembled to typical signs of PPR, but diarrhea had not a prominent sign in flocks of Navsari. As clinical signs were same in all the affected animals, only representative animals were sampled on a random basis.

Nasal swab (n = 25), mouth scab (n = 10) were collected in viral transportation medium (Earle balance salt solution with Kanamycin, pH 7.2) from live animals for antigen detection. Whereas, 25 serum samples were collected in vacutainers without anticoagulant (BD bioscience), for antibody demonstration, but one serum collection (sample no 8) leaked and could not be processed further. Necropsies were also carried out and samples were collected for histopathological examination. Utmost aseptic precautions were exercised during sample collection and were transported to the laboratory on ice. These were reached to laboratory within 3 h of collection.

### Tests carried out for diagnosis

OIE manual [2] guidance was taken in to reference and decided to carry out AGID as well as antibody demonstration ELISA at our laboratory. While at the same time samples were also sent to National Institute of Veterinary epidemiology and Disease Informatics (NIVEDI), Bangalore for antigen detection and confirmation of our results. Hence, a commercial competitive ELISA kit was ordered (IDvet, cELISA kit), meanwhile an iELISA had been developed with available resources using PPR vaccine (procured from Indian Veterinary research Institute) as antigen.

### AGID test for antibody demonstration

The test was carried out to detect antibodies against PPR virus as per Khan *et al*. [13] and OIE manual [2]. Briefly, 1% agarose suspension was made and poured in 6 ml quantity in 35 mm Petri plates. Wells were cut with cardboard template which had been supplied for 35 mm Petri plate with some other commercial AGID kit. PPR vaccine was used antigen source and serum of 45 days vaccinated goat was used as positive control with phosphate buffered saline as a negative control. Samples were placed in alternate wells and development of precipitation line within 72 h was to be recorded as a positive result. Test was declared negative if no visible line developed after acetic acid washing (5% glacial acetic acid for 5 min).

### Antibody capture iELISA

A total of 24 samples were tested with an in house developed iELISA considering earlier works [1,14]. Two 96 well flat bottom maxisorp microtiter plate (Nunc, Denmark) were coated with very high amount of vaccine antigen, where vaccine vial was reconstituted to 100 doses in 1 ml carbonate bicarbonate buffer (pH-9.4). 100 ml of suspension was put in each well. Each plate was kept overnight at 4°C at static condition. Next morning unbound antigen was washed with washing buffer (0.025% Tween-PBS, three washing of 3 min each). The wells were blocked with 200 ml 3% bovine serum albumin (BSA) in washing buffer and washed again. Serum samples in three dilutions, i.e., 1:10, 1:20 and 1:40 were applied to each well in duplicate. Earlier, we thought to apply positive control of AGID as a positive control, but we used positive and negative controls of commercial c-ELISA kit. After 1 h incubation at room temperature with shaking at 300 rpm, unbound antibodies were washed. Then, G protein conjugated antibodies with HRPO (supplied along with sheep and goat Brucella antibody detection kit by NIVEDI Bangalore) was used as detector antibodies. The color reaction was observed using outpatient department tablets (5 mg for 10 ml distilled water, Thermo Fischer scientific) with 3% H_2_O_2_. After stopping with 1M H_2_SO_4_, absorbance was recorded using 492 nm filter on ELISA reader (Ms Tecan corp). A positive-negative (P/N) ratio 2 or above [1] was considered positive upon the linearity of negative controls.

### Competitive ELISA (c ELISA)

As commercial cELISA kit (M/S IDvet, France) had been received meanwhile, laboratory confirmation of iELISA results and disease diagnosis was made through it. The test was carried out as per manufacturer instructions. Briefly, Antigen coated wells were applied with serum samples (1:2 diluted) and incubated for 45 min at room temperature, then plates were washed thrice with washing buffer. Then, HRPO conjugated antibodies directed against nucleoprotein of virus was applied to wells and again incubated for 30 min to cover unbound antigens. Washing was done again and TMB substrate solution was incubated for 15 min and reading was taken at 450 nm on ELISA reader after inclusion of stopping solution (Multisakn Ex, Thermo Corp).

### Antigen detection and histopathology

Nasal swabs, triturated mouth swabs, and tissue samples (with sterile PBS in sterile pastel and mortar) were cleared by centrifugation at 5000 rpm for 10 min and supernatants were sent to NIVEDI, Bangalore. The results received were compared and used to confirm the disease. The histopathological examination was carried out.

### Statistical analysis

Chi square test was applied as per the procedures of Snedecor and Cochran [15] to determine the significance of difference in mortality pattern between sheep and goat by PPRV infection p<0.05 and degree of freedom = 1.

## Results

### Clinical picture and statistic

The clinical signs were strongly indicative of PPR in two outbreaks, with morbidity of 100% and mortality were 73.68% (n = 392/532) and 56.67% (n = 51/90) in Vapi and Navsari outbreaks, respectively. Further, partitioning could be made in Vapi outbreak, where 81.60% (n = 315/386) goat and 52.74% (77/146) sheep were died. Significant difference was observed in disease sensitivity pattern of two species (Chi-square value = 30.49 at p<0.05, df = 1). Summary of outbreak is described in Table-1.

**Table-1:** Summary of outbreak and diagnostic tests.

Attributes	Description
Places of occurrence	Vapi (Dist Valsad) and Vansda (Dist Navsari)
Animals affected	532(Vapi)+90 (Vansada)=622
Species affected	Goat (476)+Sheep (146)
Mortality (percent)	392/532 (73.68%)+51/90 (56.67%)=71.22%
Sample collected	PM samples, Nasal Swabs (25) Mouth Swabs (10) and Sera (25)
Positive in AGID	Nil
Positive through iELISA	22/24 (Specificity 100%; Sensitivity 91.66%)
Positive through cELISA	24/24 (Specificity 100%; Sensitivity 100%)
Other tests carried out	Sandwich ELISA and histopathology

cELISA=Commercial ELISA, AGID=Agar gel immunodiffusion, iELISA=Indirect ELISA

### AGID and iELISA

The one prerequisite of applying antibody demonstration test is that there must be a negative history of vaccination and these animals should not be vaccinated with PPR vaccine. Of the 24 sera sample tested by AGID, all showed negative results for the presence of precipitation line; therefore, this test was not found useful to apply for this purpose.

### iELISA and cELISA

**Plate-1:**
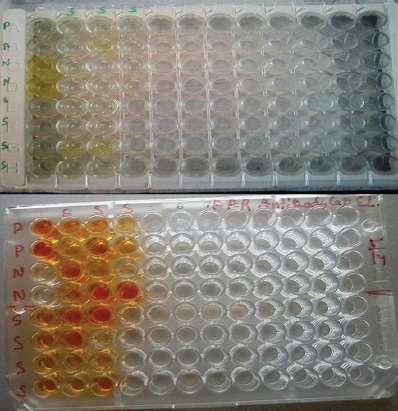
The results of ELISA tests: Upper panel is competitive ELISA with all samples wells positive and lower panel depicts indirect ELISA, two negative wells in iELISA are C4 and F3. In both plates, P and N are negative and positive controls, respectively, in duplicates.

Initially three dilutions, 1:10, 1:20, and 1:40 were used and we found that there were no difference in negative and positive wells at 1:40, whereas 1:20 gave arbitrary results and satisfactory results were obtained with 1:10 dilution and taken as final concentration. On all three dilutions, negative well showed same OD value. At 1:10 dilution, 22/24 (91.66%) sera showed positive results at P/N ratio 2. When sera were tested with commercial cELISA, all 24 serum samples showed positive results as per manufacturer cut off value. Figures of both tests are depicted with Plate-1.

### Antigen demonstration and histopathology

On the basis of the results received from NIVEDI, the outbreak was further confirmed as caused by PPRV through antigen demonstration sELISA test. Typical histopathological lesions of PPRV infection were also observed in lung, spleen, lymph nodes, and intestines during histopathological examinations. Histopathological examination of intestine revealed intracytoplasmic inclusion bodies in necrotic glands, necrotic villi, congestion, hemorrhages, and infiltration of inflammatory cells in lamina propria and depletion as well as rarefaction of lymphoid cells in Peyer’s patches. Depletion of lymphocytes was also noted in lymph nodes and spleen. Lungs revealed bronchointerstitial pneumonia, edema, hemorrhages, infiltration of mononuclear cells, and thickened interalveolar septa.

Result of immunocapture ELISA and histopathology were used to confirm the disease as PPR, not for comparison with antibody-based iELISA or cELISA. These may be discussed elsewhere by respective authors.

## Discussion

PPR is a highly contagious disease of small ruminants, affecting small ruminants mainly goat and sheep. The relative species sensitivity is usually recorded when two species are simultaneously affected. In our study, more deaths in goats compared to sheep was not an unusual finding which was also recorded previously by many workers [16,17] though few recorded goat and sheep are equally prone [18]. But, as in flocks of Navsari, diarrhea was not a prominent sign, which might skew the clinician and show the importance of laboratory diagnosis.

The high morbidity and mortality associated with PPR compel laboratory personnel to provide rapid diagnosis. On the other hand, a non-specialized laboratory, which routinely does not handle PPR may faces many problems for giving rapid and accurate diagnosis, as with any other viral diseases [19]. As a simplest available test for PPR diagnosis [2] AGID was carried out. However, in the absence of hyperimmune sera in required quantity, it was modified for antibody detection [13]. However, it did not served the purpose, possible explanation for the negative AGID result is low sensitivity of precipitation test [2]. On the other hand, sera belonged to acute cases there would have been IgM class of antibodies whereas IgG are better suited antibodies for precipitation or Immunodiffusion type of tests. Khan *et al*. [13] reported seroconversion of AGID positive antibodies after 14^th^-day post PPR vaccination, which means, it could not be a suitable test to detect early infection through antibody demonstration.

Hence, another economical test method was required and in the face of Rinderpest eradication and absence of vaccination history we thought that antibody detecting could simulate the specificity of antigen or nucleic acid-based techniques. Most of the antibodies based detection relied upon the use of cELISA either developed in house [7] or commercially procured [20]. In house, development requires generation of monoclonal antibodies [6] and conjugation step of detector antibody which are very technically demanding. Commercial kits are very costly and keeping quality of components is very poor these kits usually expire in near about a year of manufacturing. The main cause is lesser availability and fluctuation of power; this has also been ascribed to improper storage [21]. An indirect ELISA can be a suitable alternative for labor intensive and technically demanding VNT [7] and costly cELISA [1].

Regarding the development of iELISA test some modifications were applied to suit early detection of disease in the absence of validated components. As rapid results were required, instead of using checkerboard for antigen and antibody dilutions, we applied concentrated form of antigen to cover most of the sites of wells; further blocking was done with 3% BSA to check the false positive results. Last modification was the use of Protein G conjugated detector antibody, which helped us to screen goat and sheep sera simultaneously; otherwise use of anti-species antibodies necessitated two separate tests. The idea of use of applying protein G conjugated was obtained from Neilson *et al*. [14]. Protein G is a *Staphylococcus aureus* protein and bind strongly with Fc portion of the antibody. Rather, it can be applied to detect antibody of any species with isotype detection can be switched by use of protein A or protein G.

iELISA so developed could detect serum diluted to 1:10, recently, Truong *et al*. [22] attempted similar test with antigen derived from Vero cell culture and reported use of 1:50 as initial dilution where they get detectable IgG on 8^th^ day post infection. The difference is again due to the early collection of serum, which might contain low IgG level which remained undetectable at higher dilutions. However iELISA, unlike AGID can detect early disease. The iELISA proved specific in comparison of cELISA, but could not detect two serum samples as positive which was detected by commercial cELISA. The similar results have been described by Balamurgan *et al*. [1], where they reported 95.09 and 100% specificity whereas 90.01% and 80% sensitivity against cELISA and VNT, respectively. The false negative results may require correction of lower limit of detection or else there may be interference by large sized IgM in binding with antigen. On the application level, all the positive samples may be declared positive, but negative sample need to be reconfirmed with more sensitive test. As the test shown similar OD value for negative control, false positive should not be a problem.

## Conclusions

PPR is a disease of high morbidity and mortality that affect small ruminants reared by poor and marginal farmers of India. The clinical picture may vary and diarrhea may not be a prominent sign. As most of the flocks are unvaccinated against PPR virus, so an economical antibody-based test may serve the purpose of PPR diagnosis. Though, AGID was found unsuitable but an in house iELISA was proved equally specific with commercial cELISA, but showed few false negative results. sELISA and histopathological examination proved useful adjuncts for the final declaration of the outbreak as PPR.

## Authors’ Contributions

IHK and KKS designed the study. KKS, DPK, and DRP collected the samples and performed the experiments. PDV and JMP carried out necropsy and evaluated gross and histopathological changes. KKS and DPK analyzed the data. KKS, DPK, and IHK drafted and revised the manuscript. All authors read and approved the final manuscript.
